# Magnetic hydrogel scaffold based on hyaluronic acid/chitosan and gelatin natural polymers

**DOI:** 10.1038/s41598-024-78696-6

**Published:** 2024-11-15

**Authors:** Ashraf Abou-Okeil, Rakia Refaei, Shaimaa E. Moustafa, Hassan M. Ibrahim

**Affiliations:** 1 National Research Centre, Textile Research and Technology Institute, 33 El Bohouth St., Dokki, Cairo, P.O.12622, Egypt; 2 National Research Centre, Textile Research and Technology Institute, 33 El Bohouth St., Dokki, Cairo, P.O. 12622, , Egypt

**Keywords:** Magnetic hydrogel, Scaffold, Iron oxide, Chitosan, Gelatin, Hyaluronic, Drug discovery, Materials science, Nanoscience and technology

## Abstract

Owing to their native extracellular matrix-like features, magnetic hydrogels have been proven to be promising biomaterials as tissue engineering templates In the present work, magnetic hydrogels scaffold based on chitosan, gelatin, hyaluronic acid, containing Fe_3_O_4_ as magnetic nanoparticles (IONPs) were prepared. The prepared hydrogels were loaded with ciprofloxacin hydrochloride as a model drug. The magnetic hydrogel was prepared using different volumes of chitosan, 1%, gelatin, 10%, and hyaluronic acid, 1% in glutaraldehyde as the crosslinking agent and Fe_3_O_4_ as magnetic nanoparticles. The hydrogel scaffold and magnetic scaffold hydrogel samples were characterized by scanning electron microscopy (SEM), vibrating sample magnetometry (VSM), and Fourier-transform infrared spectroscopy (FTIR). The porosity, mechanical properties, swelling degree, and antibacterial activity of the hydrogel scaffold were also determined as well as the drug release profiles of the hydrogels. SEM imaging revealed that the magnetic hydrogel scaffold showed a relatively rough morphology with an irregular surface. The data obtained indicated that the hydrogel surface has three-dimensional porous microstructures and the porosity varied depending on the hydrogel formulation. The breaking load of the hydrogel scaffold increased from 1.361 Kgf to 4.98 Kgf by increasing the glutaraldehyde concentration from 0.2 mL to 0.8 mL. Swelling degree values in water were from 250 to 2000% after 24 h. The antibacterial activity of the hydrogel scaffold ranged from 54% to about 97% for Gram-positive bacteria (*S. aureus*) and from about 26–92% for Gram-negative bacteria (*E. coli*). The ciprofloxacin hydrochloride loaded hydrogel has a sustained release of ciprofloxacin hydrochloride over 10 h. The presence of IONPs gave a faster release of ciprofloxacin hydrochloride.

## Introduction

Hydrogels are hydrophilic polymers made of three-dimensional viscoelastic networks that can hold many times their dry weight in water and swell in physiological conditions^1,2^. Hydrogels’ physical interactions and chemical cross-linking *can*help maintain their structural and physical integrity. They are used as vendors in the drug delivery system^3–5^. Hydrogel properties such as porous media and their shapes and particle size distribution have significant effect on the prepared hydrogels. Biomaterials are supposed to interact with biological structures to treat, augment, or regenerate tissue, organ, of the body^6,7^.

The use of gelatin in pharmaceutical and biomedical disciplines is extremely attractive because it is non-toxic, biodegradable, inexpensive, non-immunogenic, and has an excessive capacity to be used in many medical applications. It has additionally been used as a wound dressing^8–11^, hemostatic materials^12–15^, sealant for vascular prosthesis^16,17^, drug delivery systems together with tough and gentle capsules^18,19^, hydrogels^20^, or microspheres^21–23^and finishing agent^24^. The benefits of the use of gelatin over collagen encompass its water solubility and lesser cost. Because of their properly film-forming abilities, gelatin can be a good replacement to artificial plastics for making films to maintain food-stuffs^25^. Gelatin gel suggests non-cytotoxicity towards human cells and is biodegradable in nature^25,26^.

Chitosan is a natural and widely used polysaccharide composed of glucosamine and N-acetylglucosamine^27,28^. It is made from chitin, which is derived primarily from crustacean exoskeletons^29^. It has been widely used in a variety of biomedical fields, including drug delivery, gene delivery, wound healing, cell imaging, surgical sutures, sensors, wastewater management, tissue engineering, antibacterial agents, food processing, tissue engineering medicine, and biotechnology. Chitosan’s biodegradability, bio-renewability, biocompatibility, muco-adhesiveness, antibacterial, hemostatic, cell compatible, and low toxic properties have made it one of the interesting advanced materials that have found broad applicability, not only in the biomedical also in the conventional pharmaceutical domain too^30^.

Hyaluronic acid, or hyaluronan (HA), is a biopolymer that can be modified and processed to create hydrogels for biomedical applications^31^. Because of their biocompatibility, tunable properties, and native bio functionality, HA hydrogels are becoming more versatile for a wide range of applications^31^. HA has inherent biological importance because of its ability to bind to receptors such as CD44, degrade via oxidative species and hyaluronidases, and play a role in development, wound healing, and adult tissue function and structure^32^. With these characteristics in mind, several HA hydrogel systems have already been clinically tested in human and veterinary patients, particularly as dermal fillers, intra-articular supplements, corneal and dermal wound repair, and post-surgical adhesion prevention^33^.

Ciprofloxacin is one of the antibiotics with bactericidal activities in the case of bone infection (osteomyelitis). Ciprofloxacin is the most commonly used fluoroquinolone for bacterial bone infections due to its low minimal inhibitory concentration (MIC) (0.25–2 µg/ml) for most pathogens that cause osteomyelitis, including Staphylococcus aureus, Staphylococcus epidermidis, Pseudomonas aeruginosa, and Proteus mirabilis. Biomaterial implants that operate as a ciprofloxacin-controlled delivery system could be developed as an alternate treatment for osteomyelitis. Biomaterials used to build implants must be biocompatible, biodegradable, osteoconductive, angiogenic, and have mechanical strength to support the formation of bone tissue^34^.

Recently, because nanotechnology has emerged, diverse kinds of nanomaterials have been included in biopolymer-primarily based hydrogels to improve the physicochemical properties of the hydrogels. For example, gold nanoparticles had been integrated into a carrageenan-primarily based totally hydrogel for optical belonging modulation and managed the discharge of drugs^35,36^. , silver and magnetic nanoparticles (Fe_3_O_4_) had been used to increase growth, increase swelling efficiency, and improve drug transport systems^37^. , sodium montmorillonite (Na-MMT) nanoclay has been used to improve the dye adsorption capacity^38^. , and nanoclay and silver nanoparticles had been incorporated into carrageenan-primarily based totally films to have UV barrier and antimicrobial residences^39^.

Magnetic hydrogels with order structure, which are like in structure to the native mechanical microenvironment in vivo, are better suited for cell culture than ordinary hydrogels. For example, a sliced magnetic hydrogel was used with anisotropic design as a 3D cell culture substrate^40^. Even in a traditional 3D cell culture setup, cells could spontaneously form multicellular spheroids instead of loose aggregates. Magnetic hydrogels with ordered structures have also been employed in tissue regeneration to mimic native tissues and initiate their natural reparative process, including applications in nerves, skin, cartilage, and muscles^41,42^. As neural regeneration scaffolds, hydrogels can control neuronal development, causing neurons to form coordinated morphologies. Hydrogels with variable microstructure orientations can be generated by varying the magnetic field for diverse tissue engineering applications. Silica rods coated with magnetic nanoparticles was used to impart orientational order to collagen hydrogels under a magnetic field^41^. A small magnetic field can easily align these extremely anisotropic nanorods, influencing the development of normal human dermal fibroblasts. A recent study used magnetic collagen-based hydrogels with directed structures to drive growth. Human adipose stem cells were studied^43^, the actin filaments of human adipose stem cells are aligned with collagen fibers and MNPs. The structures may mimic both normal and diseased tissues, indicating a feasible strategy for tendon healing. Developed anisotropic collagen-based substrates were used to direct the growth of human mesenchymal stem cells^44^.

Owing to their native extracellular matrix-like features, magnetic hydrogels have been proved to be promising biomaterials as tissue engineering templates^45,46^. Many biological tissues present a specific organization of ordered structures, which is important for physiological functions^41^. Magnetic hydrogels with ordered structure can provide a template for directional growth of cells and control their behavior, which have attracted extensive researches^46^. The aim of the present work is to synthesize and characterize a magnetic hydrogel scaffold with ordered structure based on natural polysaccharides to be used as a template for directional growth of cells and control their behavior. The magnetic hydrogel scaffold will be loaded with ciprofloxacin hydrochloride as a model drug. The loading and release profiles of ciprofloxacin hydrochloride as well as the antibacterial properties of the magnetic hydrogel scaffold were also evaluated.

## Materials and methods

### Materials

FeCl_3_·6H_2_O and FeCl_2_·4H_2_O were purchased from Merck (Darmstadt, Germany). NaOH (25 wt% in water).  Hyaluronic acid (HA) (average molecular weight 1.48*10^6^ Da), supplied by Acros Organics was used. Chitosan of molecular weight (MMW, 480 kDa) and degree of deacetylation of 79.0% (Alfa Aesar Company) were used. Glutaraldehyde (50%) and ciprofloxacin hydrochloride were purchased from Fisher Company and used without further purification. Acetic acid and gelatin are of laboratory-grade chemicals. All chemicals were used without any further purification.

### Methods

#### Synthesis of Fe_3_O_4_ magnetic nanoparticles (IONPs)

**Fig. 1:**
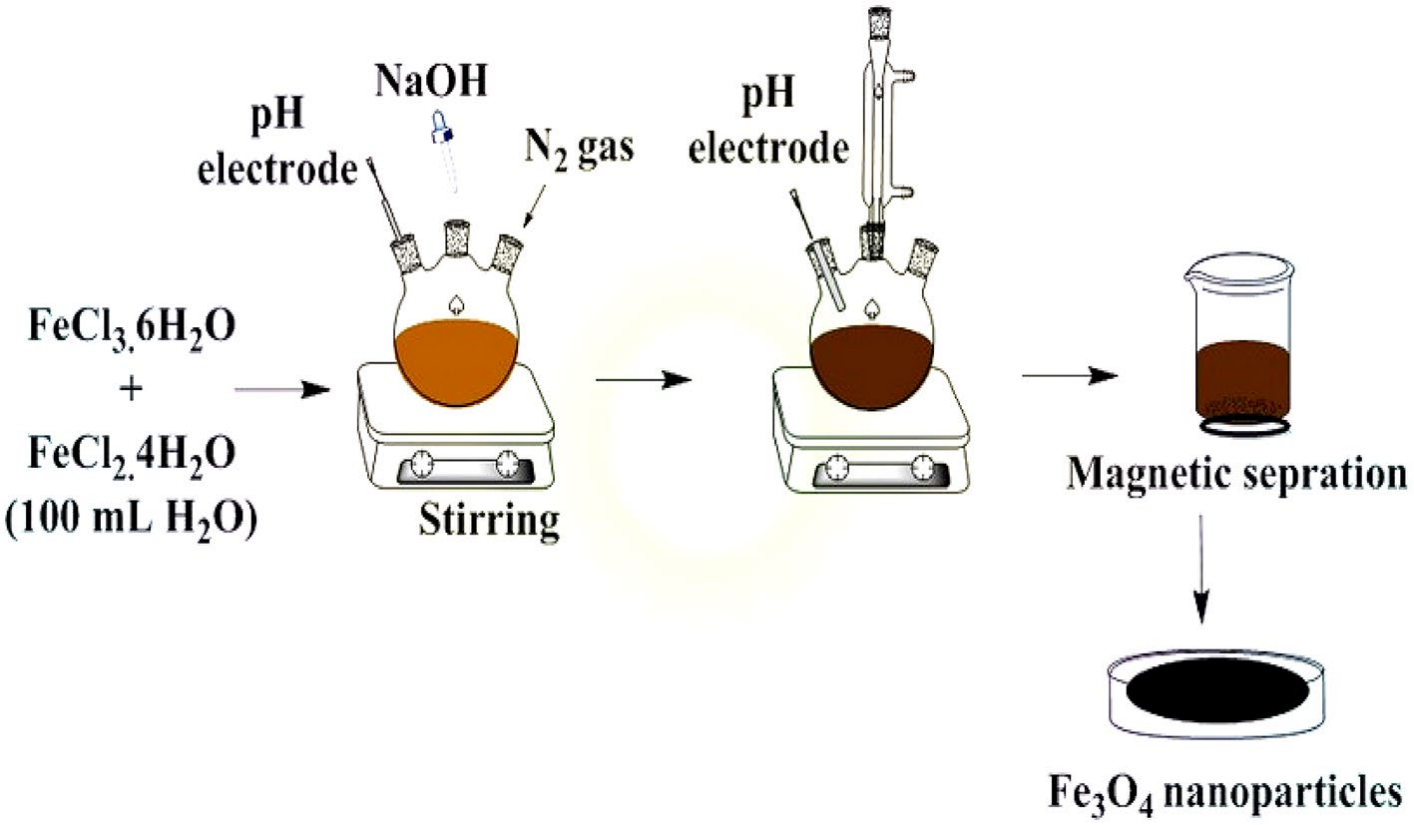
Schematic diagram of synthesis of Fe_3_O_4_ magnetic nanoparticles.

**Fig. 2:**
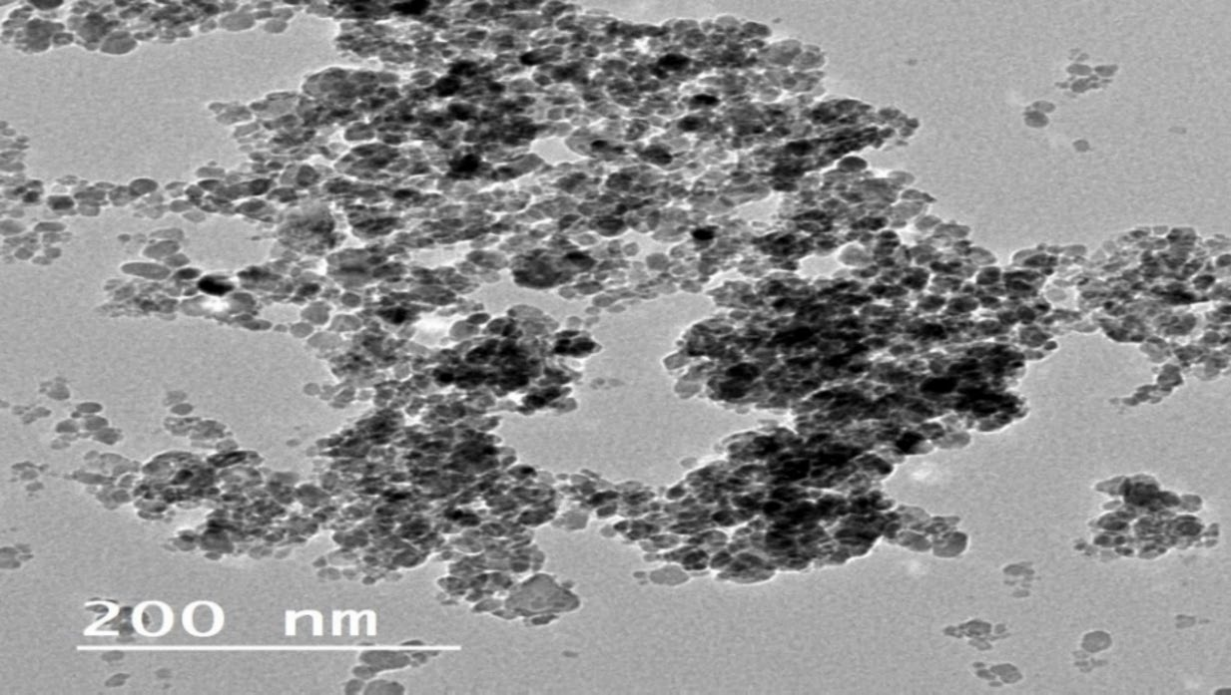
TEM image of Fe_3_O_4_ magnetic nanoparticles.

An aqueous solution of FeCl_3_·6H_2_O (1.1 g) (0.0037 mol) and FeCl_2_·4H_2_O (0.4 g) (0.002 mol), in the molar ratio 2Fe (III): 1Fe (II) in 100 mL in deionized water was prepared and kept at a constant temperature of 60^◦^C for 15 min (min) under vigorous stirring. Then under vigorous stirring and N_2_ gas a solution of ammonium hydroxide [20 mL NaOH (25%)] was added till the pH was reached to ∼11 at which a black suspension was formed. This suspension was then stirred at 50^◦^C for 2 h. IONPs, were separated from the aqueous solution by external magnet, washed with deionized water several times then dried at vacuum oven overnight. Figures1 and 2 show a schematic diagram of synthesis of IONPs and TEM image of the prepared IONPs, respectively.

#### Preparation of hydrogel

**Fig. 3:**
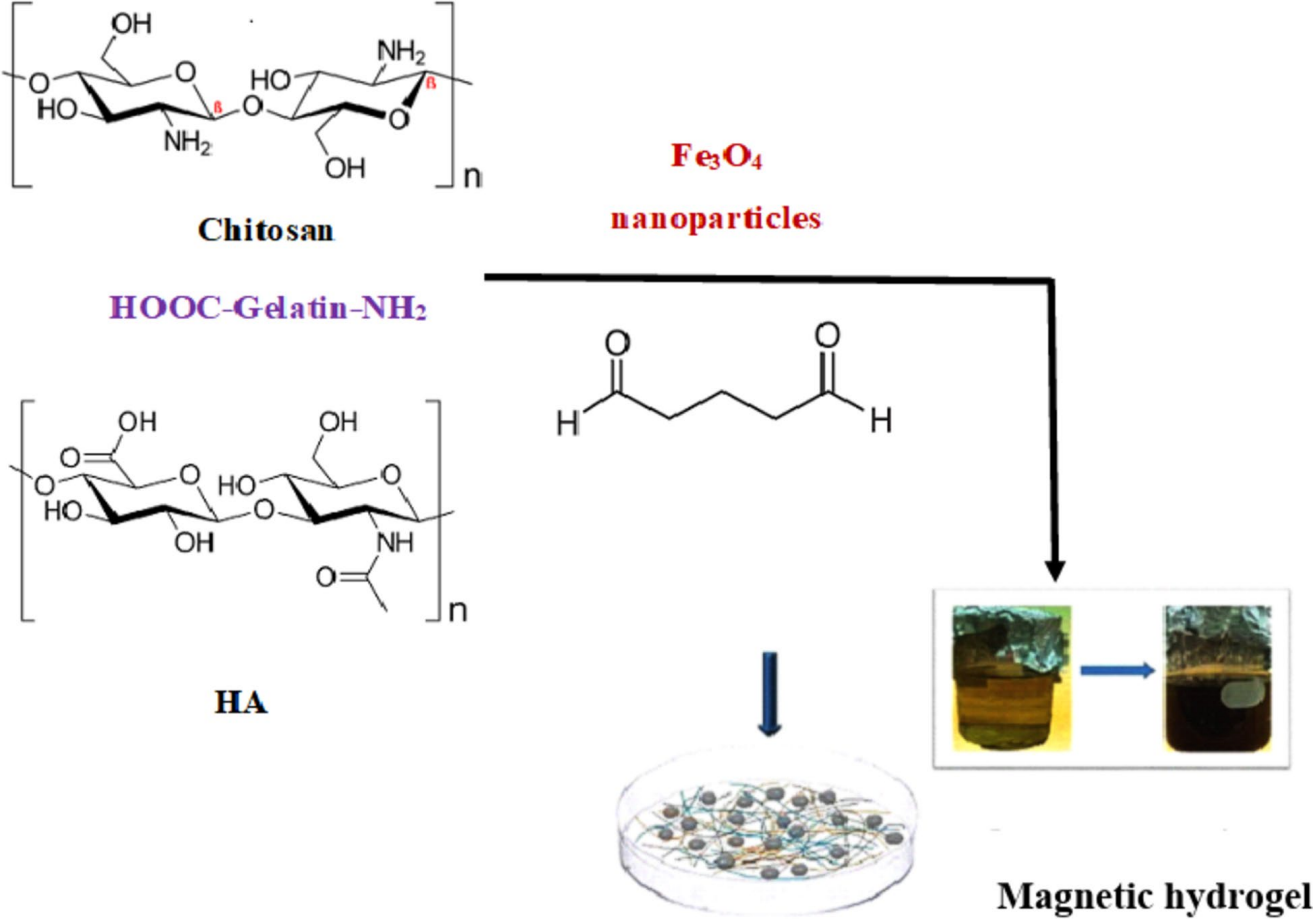
Preparation of the hydrogel using different formulations.

**Fig. 4:**
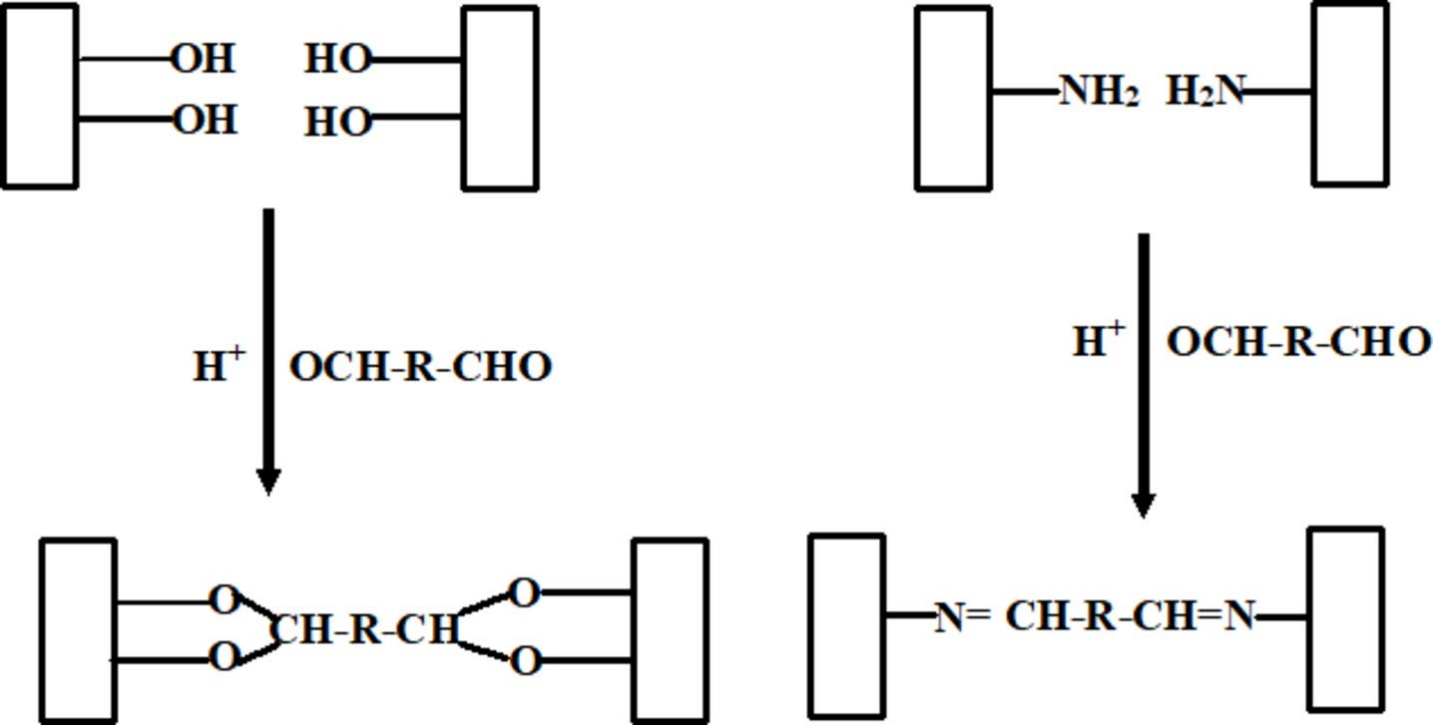
Crosslinking mechanism.

Chitosan, 1% (w/w) in 1% acetic acid, gelatin (10% w/w) in water, and HA (1% w/w) in 0.1 M HCl, solutions were prepared (Table 1) with continuous stirring until complete dissolution. Different volume ratios of each solution were added to each other’s (according to Table 1), mixed completely with continuous stirring for 1 h, then different amounts of glutaraldehyde (50%) as a crosslinking agent was added with stirring. The so obtained hydrogels were washed several times with distilled water to remove the unreacted materials, and then hydrogel samples were freeze dried and stored in desiccator until testing. The schematic diagram of the preparation process is shown in Fig. 3. And the mechanism of crosslinking is represented by Fig. 4.

**Table 1:** Preparation of the hydrogel using different formulations.

Sample code	Chitosan (1%) (in 1% acetic acid) (mL)	Gelatin (10%) (In hot water) (mL)	Glutaraldehyde (50%) (mL)	HA (1%) (In 0.1 M HCl) (mL)	H_2_O (mL)
CHT-HA-GEL 1	12	4	0.2	4	0
CHT-HA-GEL 2	12	4	0.4	4	0
CHT-HA-GEL 3	12	4	0.6	4	0
CHT-HA-GEL 4	12	4	0.8	4	0
CHT-HA-GEL 5	9	4	0.8	4	3
CHT-HA-GEL 6	6	4	0.8	4	6
CHT-HA-GEL 7	9	3	0.8	4	4
CHT-HA-GEL 8	9	2	0.8	4	5

#### Preparation of ciprofloxacin hydrochloride loaded magnetic hydrogel samples

The ciprofloxacin hydrochloride loaded magnetic hydrogel samples were prepared by blending chitosan, gelatin, and HA solutions (According to sample CHT-HA-GEL 6) (Table 1). Typically, 100 mg of ciprofloxacin hydrochloride was added in presence of 0%, 1%, 2%, and 4% of IONPs (w/w) to formulation CHT-HA-GEL 6 (Table 1), respectively to prepare CHT-HA-GEL-CIPR-IONPs 0, CHT-HA-GEL-CIPR-IONPs 1, CHT-HA-GEL-CIPR-IONPs 2 and CHT-HA-GEL-CIPR-IONPs, respectively at room temperature with stirring using magnetic stirrer, followed by adding 0.8 mL of glutaraldehyde (50%) as cross-linker. The obtained hydrogel samples were washed several times with distilled water to remove the unreacted materials. The final dried hydrogel samples were freeze dried and stored in desiccator until testing.

### Testing and analysis

1. Attenuated total reflection-Fourier transform infrared.

Infrared spectra were measured using high-resolution attenuated total reflection‐Fourier transform infrared spectroscopy (ATR‐FTIR) (JASCO FT/IR‐4700 spectrophotometer from Japan).

2. Morphology characterization.

The surface and cross-sectional morphology of freeze-dried hydrogel samples were examined by scanning electron microscopy (SEM) using the JEOL JSM-6335 F. All samples are conditioned in a desiccator for 24 h. The samples were sectioned by scissors.

3. Swelling rate (SR %).

The SR% of freeze-dried hydrogels was measured by immersion of a definite weight of the freeze-dried hydrogel in a phosphate-buffered saline (PBS) solution pH 7.2 at 37 °C. Typically, the initial weights (Wa) of the freeze-dried samples of hydrogel were determined before immersion, while the weights of the wet hydrogel (Wb) were recorded at different time intervals, reaching 24 h of incubation, after removing the surface water by smooth squeezing of the wet hydrogel with two filter papers. The swelling percentage (swelling rate) was determined by the following equation:

$$\:\:SR\%=\left[\frac{Wb-Wa}{Wa}\right]\times\:100$$

4. In vitro drug release profile.

**Fig. 5:**
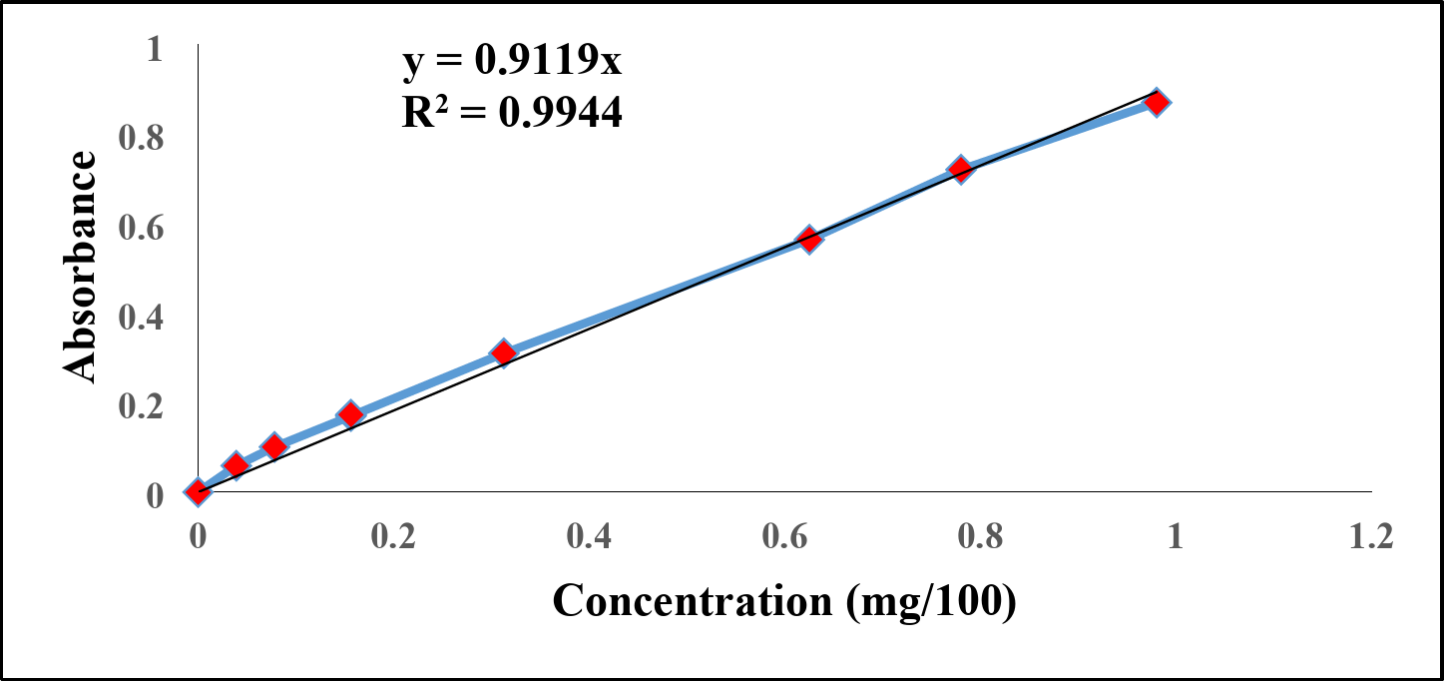
Calibration curve of ciprofloxacin hydrochloride.

The release rate of from the hydrogels was measured by incubating 0.056 g of the freeze-dried hydrogel/ ciprofloxacin hydrochloride samples in 100 mL PBS (pH 4 and 7.2) at 37 °C in a shaking water bath. At different time intervals, 2.5 mL of the medium was taken, and 2.5 mL of fresh medium was added. The absorbance was recorded at 276 nm using an ultraviolet‐visible (UV‐Vis) spectrophotometer (SHIMADZU, UVmini‐1240). The released amount was measured using the following calibration curve (Fig. 5).

5. Mechanical properties and the gelation time.

The compressive modulus of the hydrogels was evaluated using an Instron 3345, USA, equipped with a 500 kg load cell and a 152.4 mm diameter between two compression platens. The cylindrical samples of 34 mm diameter and 33 mm thickness were centered on the lower platen. The platen separation was adjusted when the installed software, Merlin, was programmed to compress the samples with crosshead platen at a speed equal to 25.4 mm/min^47^.

6. Porosity determination.

The porosity of freeze-dried hydrogel scaffolds was determined using the following method^48,49^. In brief, all the freeze-dried hydrogel scaffolds were cut into rectangular pieces of 10 mm by 20 mm. The mass and volume of each scaffold before immersing in anhydrous methanol were recorded as W1 and V, respectively. After immersion in absolute methanol for 2 h, the mass of each saturated sample was recorded as W2. Finally, the porosity of the freeze-dried hydrogel scaffolds was determined in terms of the following equation:

$$\:Porosity=\left[\frac{W2-W1}{{\uprho\:}\text{V}}\right]\times\:100$$

Where W1 and W2 are the mass of the hydrogel before and after immersion in methanol, respectively, ρ is the density of absolute methanol at room temperature, and V is the volume of the hydrogel. Five parallel samples were used for the measurement.

7. Antibacterial properties.

The antibacterial assessment was measured by the “bacterial count method” as reported elsewhere to find out the resistance of the hydrogel to different species of bacteria^50^, against the following bacteria strains:

a) “Gram-positive bacteria”: “*Staphylococcus aureus*” (*S. aureus*).

b) “Gram-negative bacteria”: “*Escherichia coli*” (*E. Coli*).

“According to that method, a liquid culture was prepared by mixing 0.5 g peptone and 0.3 g beef extract in 100 mL of water. 1 gm of the hydrogel samples was cut and put into 10 mL of liquid culture, to which 10 µL of microbe culture was added, and the tested samples were then incubated for 24 h at 37°C. From each incubated sample, 100 µL of the solution was taken, diluted, and distributed onto an agar plate. All plates were subjected to incubation for 24 hours, and the colonies formed were then counted. The percentage reduction was determined as follows:

$$\:{\%}\:Reduction\:in\:CFU\:\left(colony\:forming\:units\right)=\frac{C-A}{C}\:\times\:100$$

Where C and A are the colonies counted from the plate of the control and treated samples, respectively.

8. Vibrating sample magnetometry (VSM).

The magnetic properties were studied with a vibrating sample magnetometer (VSM). The hysteresis loops of magnetic prepared IONPs and magnetic hydrogel scaffold were investigated by the VSM technique at 298 K. The magnetization was recorded in an applied magnetic field of − 20,000 ≤ H (Oe) ≤ 20,000. The hysteresis loop and saturation magnetization were recorded.

## Results and discussion

### Preparation the hydrogel scaffold

#### FTIR

**Fig. 6:**
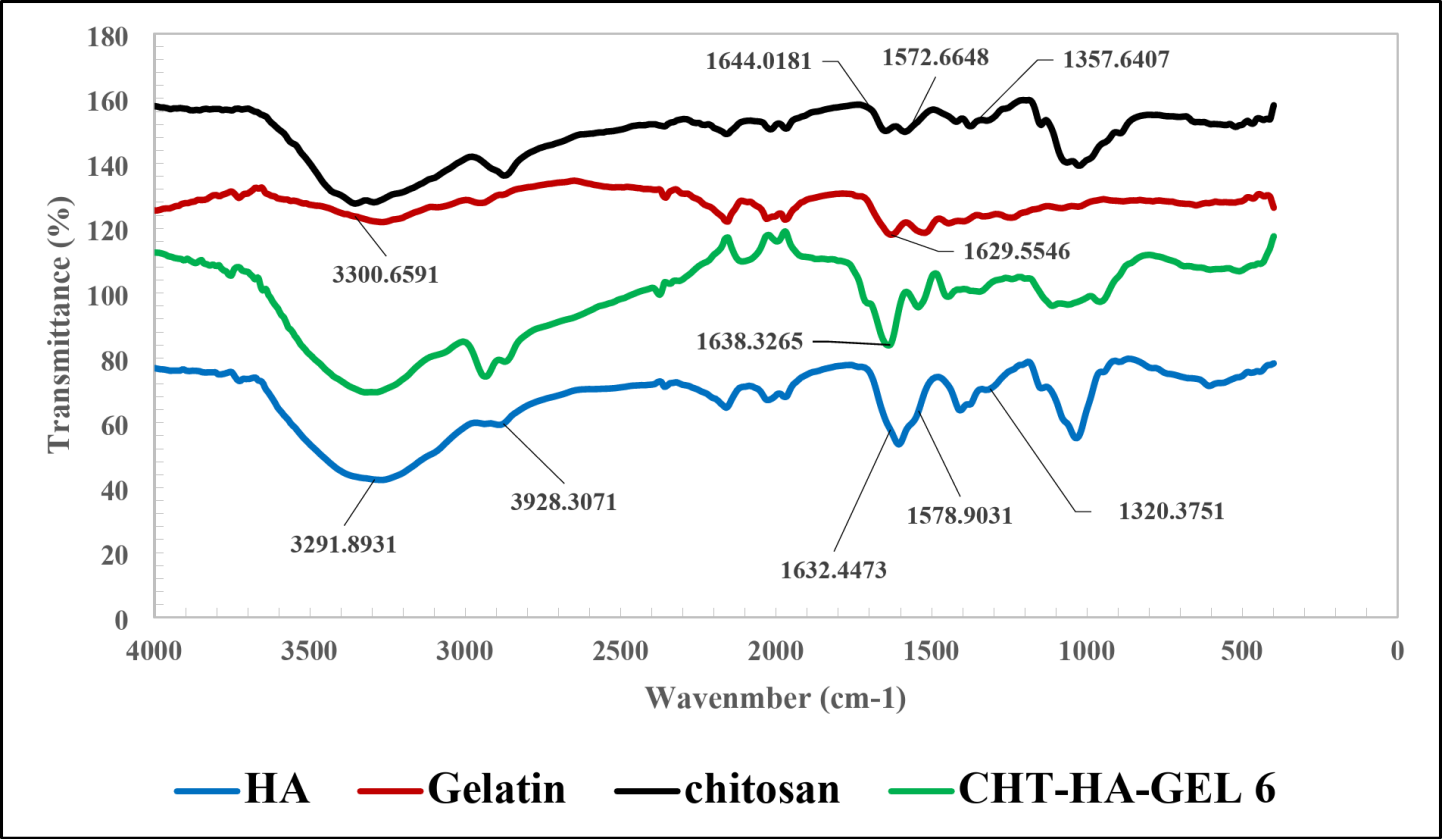
FTIR of HA, gelatin, chitosan, and CHT-HA-GEL 6.

Figure 6 shows FTIR spectra of the CHT-HA-GEL 6 and its components. The characteristic peaks of chitosan such as, C = O band at 1644 cm^−1^, NH bending band at 1572 cm^−1^ and CH bending at 1357 cm^−1^ are present. Figure 6showed also, the noticeable peaks of gelatin including absorption peak at 3300 cm^-1^ for N–H stretching, the peak at 1629 cm^-1^ refers to the absorption band of C = O^51^. FTIR spectra of HA showed the bands at 1632 cm ^− 1^, 1578 cm^-1^, and 1320 cm^-1^ correspond to amides, the band at 2928–2932 cm ^− 1^ of C-H stretching, and broadband at 3200 and 3500 cm^−1 ^because of the OH groups and N-H stretching vibration in the N-acetyl group. The major alcohol C-O stretch reached a high of 1023 cm ^− 1^^52^. Similar peaks were observed in the hydrogel prepared from chitosan, HA and gelatin in the FTIR pattern, with different intensities and peak positions in addition to the appearance of peak at 1638 cm-1 in the hydrogel which could be attributed to C = N stretching band of Schiff’s base^53^. This is can be explained by the interactions between the polymers with each other’s and also the interaction of the polymers with the crosslinking agent (glutaraldehyde)^51^.

#### Morphology of the prepared hydrogels

**Fig. 7:**
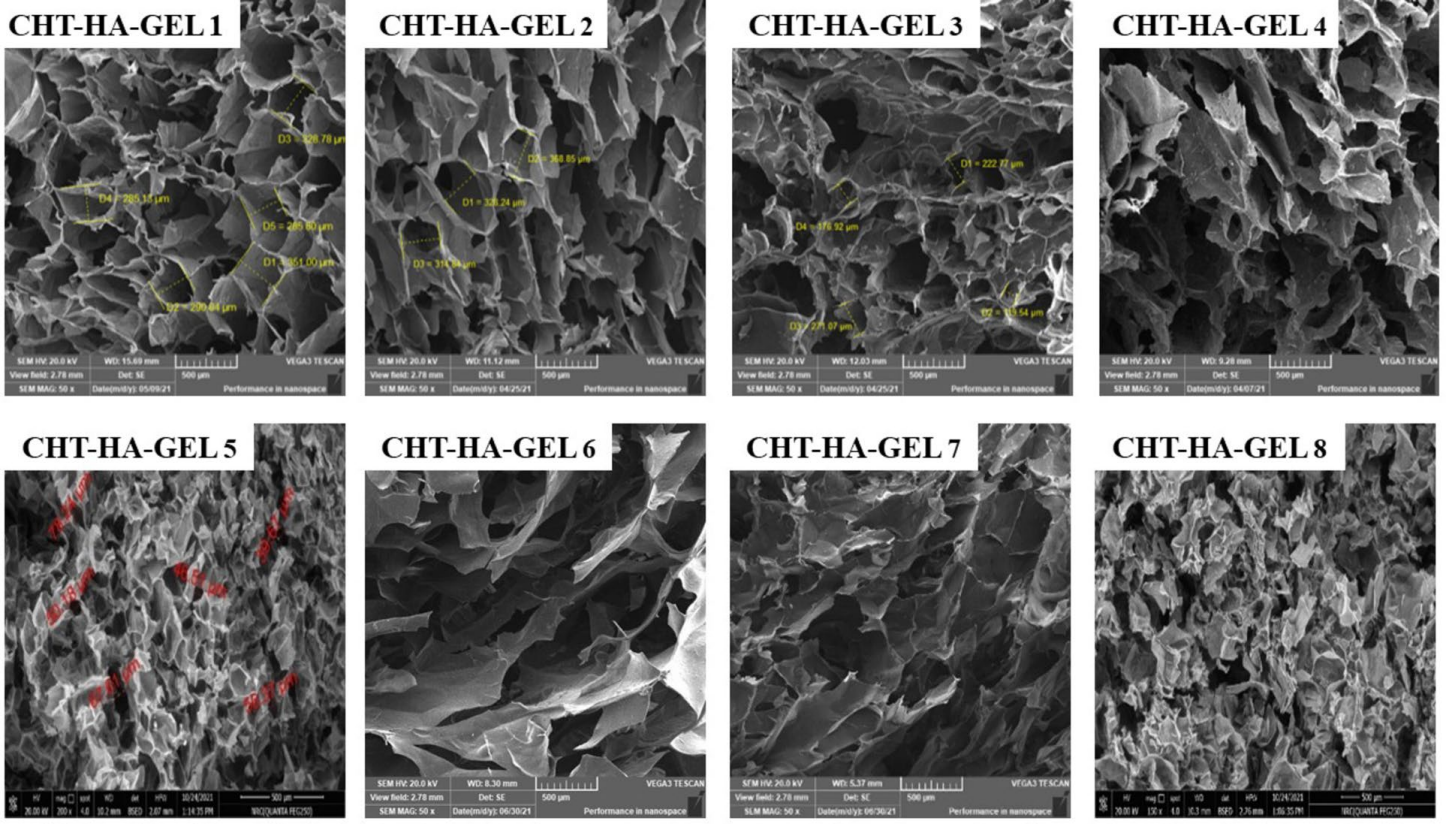
SEM images of prepared hydrogel using different formulations.

SEM images of CHT-HA-GEL 1, CHT-HA-GEL 2, CHT-HA-GEL 3, CHT-HA-GEL 4, CHT-HA-GEL 5, CHT-HA-GEL 6, CHT-HA-GEL 7, and CHT-HA-GEL 8 are shown in Fig. 7. All samples were porous and possess linked three-dimensional microstructures, as shown in Fig. 7. The creation of a cross-linked network in the gel is responsible for the connections between the pores. If these samples are utilized in tissue engineering applications, a well-connected porosity network can provide efficient nutrition and gas exchange, resulting in improved cell proliferation and survival. The pore size was in the range 60 μm to about 180 μm.

#### Mechanical properties

The mechanical properties (Table 2) of the CHT-HA-GEL 1, CHT-HA-GEL 2, CHT-HA-GEL 3, CHT-HA-GEL 4, CHT-HA-GEL 5, CHT-HA-GEL 6, CHT-HA-GEL 7 and CHT-HA-GEL 8 are the most important factor in understanding the properties of these samples. The stiffness and workability of the samples might indicate the hydrogel’s application characteristics. The mechanical properties of the hydrogel under compression were plotted in Table 2 as a function of varied glutaraldehyde concentration (CHT-HA-GEL 1, CHT-HA-GEL 2, CHT-HA-GEL 3, and CHT-HA-GEL 4). Also, at different chitosan concentrations (CHT-HA-GEL 5 and CHT-HA-GEL 6) and different gelatin concentrations (CHT-HA-GEL 7 and CHT-HA-GEL 8) (Table 1). The breaking load of these samples increased from 1.361 Kgf to 4.98 Kgf by increasing the glutaraldehyde concentration from 0.2 mL to 0.8 mL which is accepted due to the stabilization of the hydrogel structure through crosslinking process^54,55^.

**Table 2:** Mechanical properties of the prepared hydrogel.

Sample code	Breaking load (Kgf)	Maximum stress (Kgf/mm^2^)	Maximum strain (mm/mm)	Breaking strain (%)	Breaking stress (Kgf/mm^2^)
CHT-HA-GEL 1	1.361	0.0014	0.9770	97.7	0.0014
CHT-HA-GEL 2	1.9771	0.0021	0.9777	97.77	0.0021
CHT-HA-GEL 3	3.337	0.0035	0.9787	97.82	0.0035
CHT-HA-GEL 4	4.98	0.0052	0.9781	97.81	0.0052
CHT-HA-GEL 5	3.85	0.0175	1.024	102.4	0.0175
CHT-HA-GEL 6	3.9751	0.0041	1.014	101.5	0.0041
CHT-HA-GEL 7	2.624	0.0046	0.9980	99.88	0.0046
CHT-HA-GEL 8	7.7	0.0135	1.028	102.8	0.0135

As the concentration of glutaraldehyde increases the number of crosslinking bridges increases. Decreasing chitosan concentration and gelatin concentration have an inverse effect on the breaking load (Tables 1 and 2) which is logic due to the decrease of the functional groups available for crosslinking so decreasing the extent of crosslinking and by the way decreasing the stability of the hydrogel samples.

#### Porosity

**Fig. 8:**
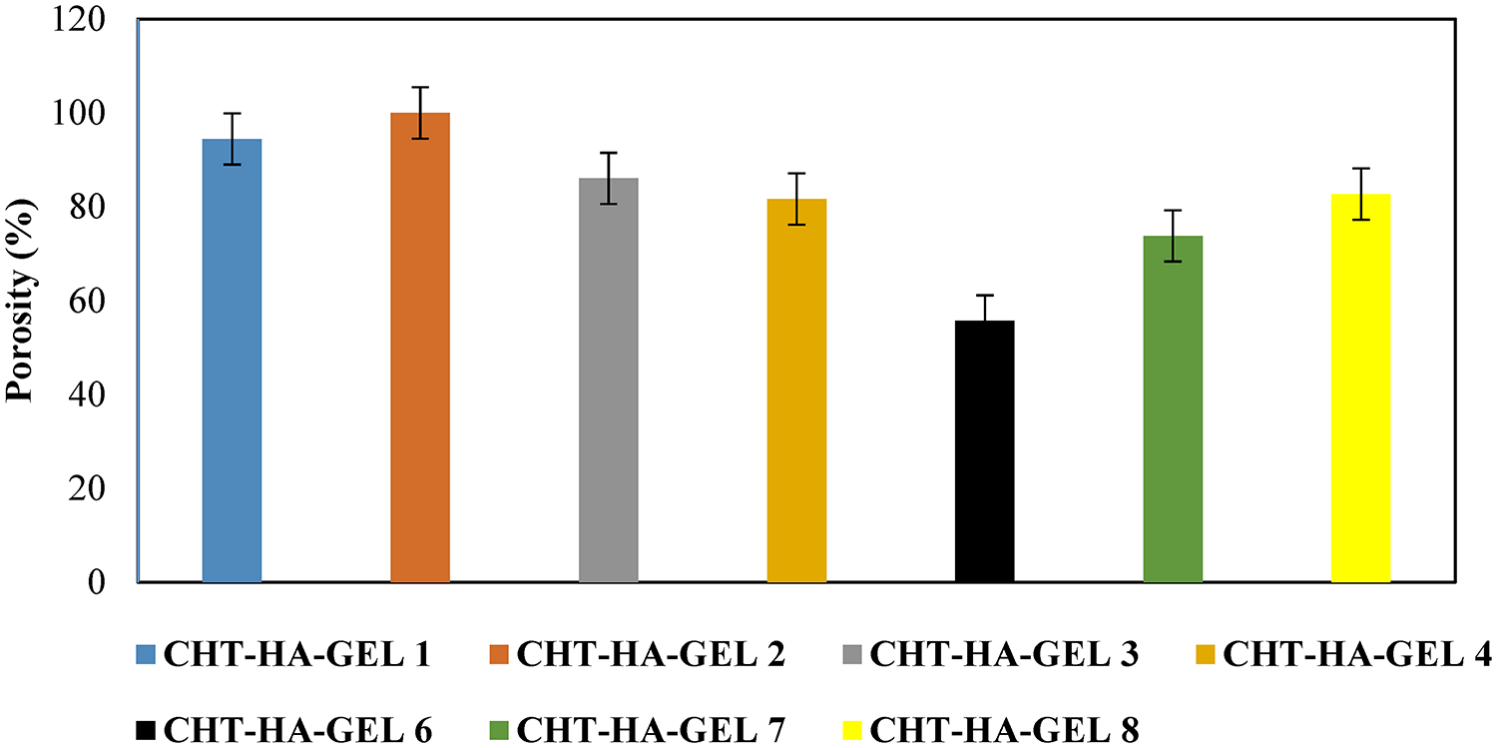
Porosity % of the prepared hydrogels.

Figure 8 illustrates the porosities of CHT-HA-GEL 1, CHT-HA-GEL 2, CHT-HA-GEL 3, CHT-HA-GEL 4, CHT-HA-GEL 5, CHT-HA-GEL 6, CHT-HA-GEL 7, and CHT-HA-GEL 8. It can be seen that the porosity varied depending on the hydrogel composition used. The increase in porosity could be attributed to a rise in the viscosity of the solution used to make the hydrogel, which effectively prevents bubbles from escaping. This phenomenon causes the porosity to increase and the creation of linked channels. The pore size was in the range 60 μm to about 180 μm.

#### SR %

**Fig. 9:**
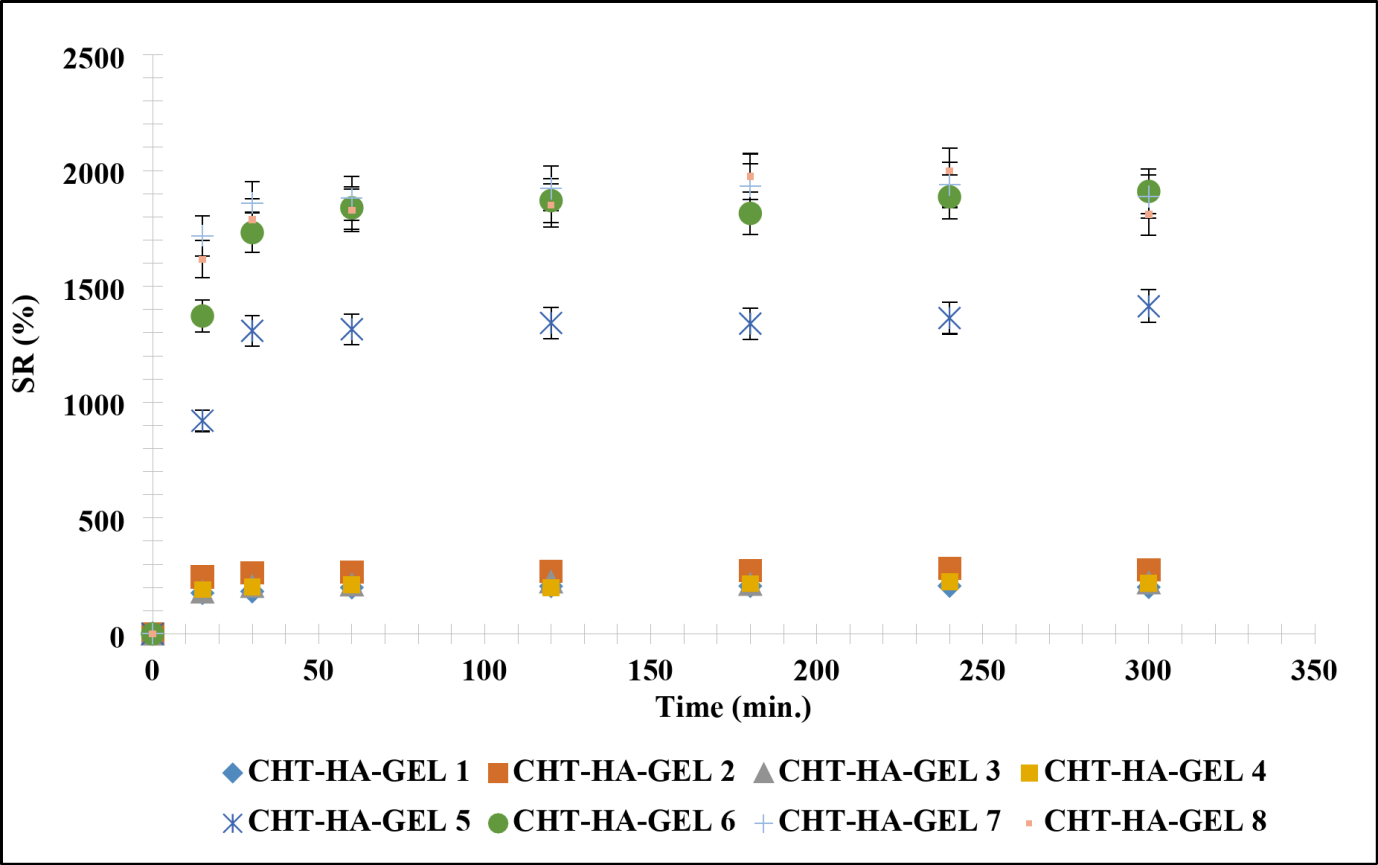
Swelling rate of the prepared hydrogels samples.

Figure 9 shows SR % of CHT-HA-GEL 1, CHT-HA-GEL 2, CHT-HA-GEL 3, CHT-HA-GEL 4, CHT-HA-GEL 5, CHT-HA-GEL 6, CHT-HA-GEL 7, and CHT-HA-GEL 8. The SR% of the hydrogel increased significantly within the first 1 h of immersion in water, and then gradually increased until 24 h of immersion, when it stabilized. At the equilibrium samples have its maximum SR%.

#### Antibacterial properties

**Fig. 10:**
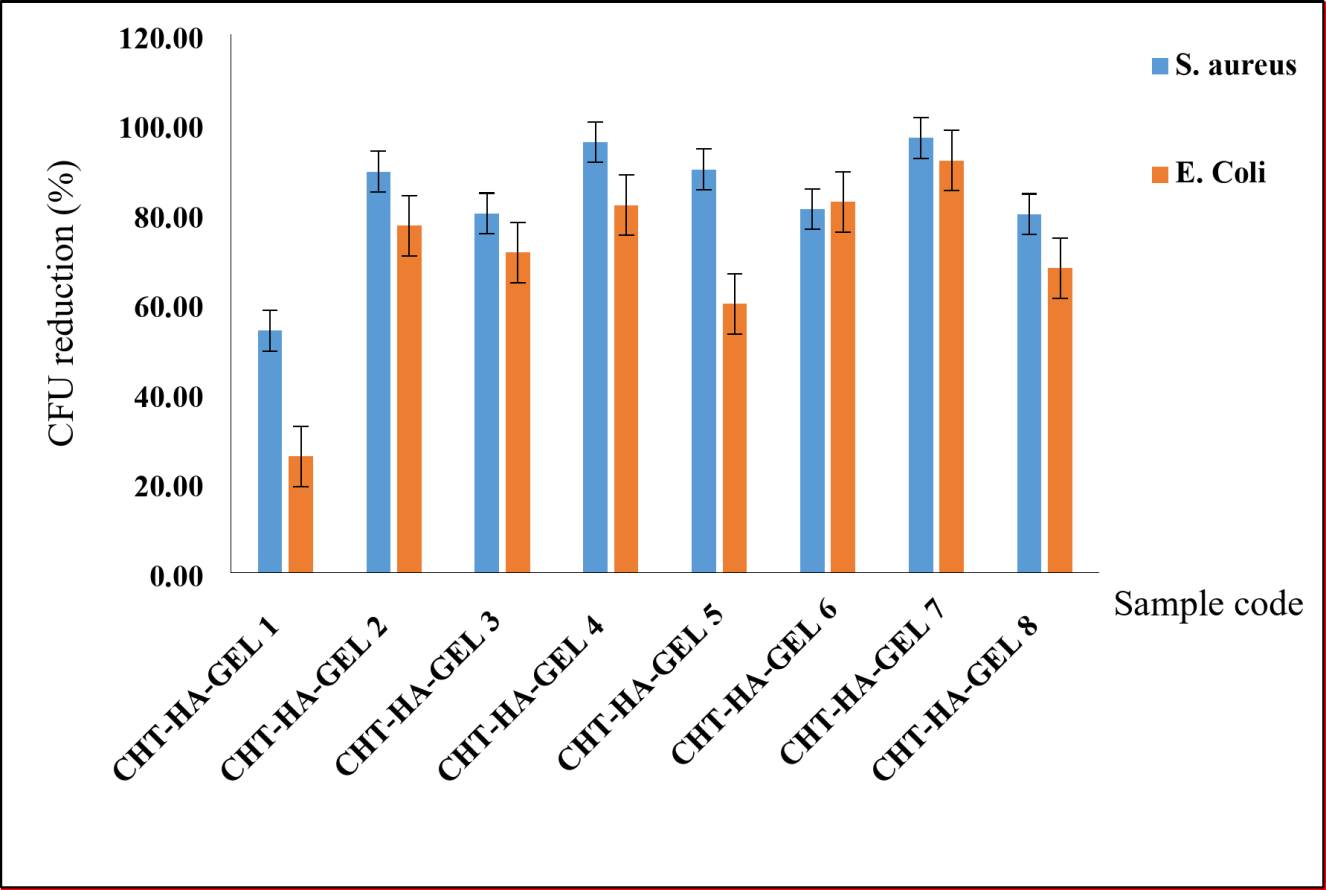
Antibacterial activity of various hydrogel samples.

The antibacterial activity of CHT-HA-GEL 1, CHT-HA-GEL 2, CHT-HA-GEL 3, CHT-HA-GEL 4, CHT-HA-GEL 5, CHT-HA-GEL 6, CHT-HA-GEL 7 and CHT-HA-GEL 8 is depicted in Fig. 10 as a percent reduction in CFU of *Gram-positive (S. aureus) and Gram-negative (E. coli)* bacteria. As illustrated in Fig. 10, all hydrogels’ samples synthesized exhibit a strong antibacterial activity against *Gram-positive (S. aureus) and Gram-negative (E. coli)* bacteria. The antibacterial activity of the hydrogels samples was ranged from 54% to about 97% for *Gram-positive bacteria* (*S. aureus*) and from about 26–92% for *Gram-negative* bacteria (*E. coli*). Increased chitosan content enhances the antibacterial activity of both *Gram-positive (S. aureus)* and *Gram-negative bacteria (E. coli*).

### Preparation of magnetic hydrogel

#### SEM

**Fig. 11:**
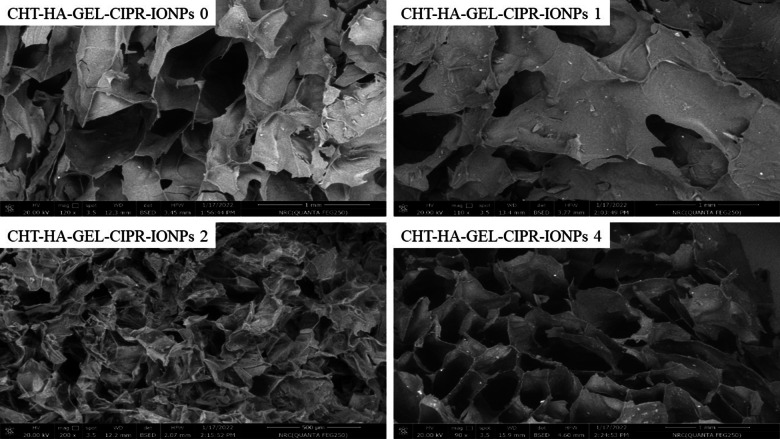
SEM images of prepared hydrogel with different amount of magnetic nanoparticles.

Figure 11. shows SEM images of freeze-dried CHT-HA-GEL-CIPR-IONPs 0, CHT-HA-GEL-CIPR-IONPs 1, CHT-HA-GEL-CIPR-IONPs 2, and CHT-HA-GEL-CIPR-IONPs 4. SEM images revealed that the freeze-dried samples surface was relatively irregular, with a granulated layer covering the entire sample, which was most likely caused by the crosslinking agent (Fig. 11).

#### VSM

**Fig. 12:**
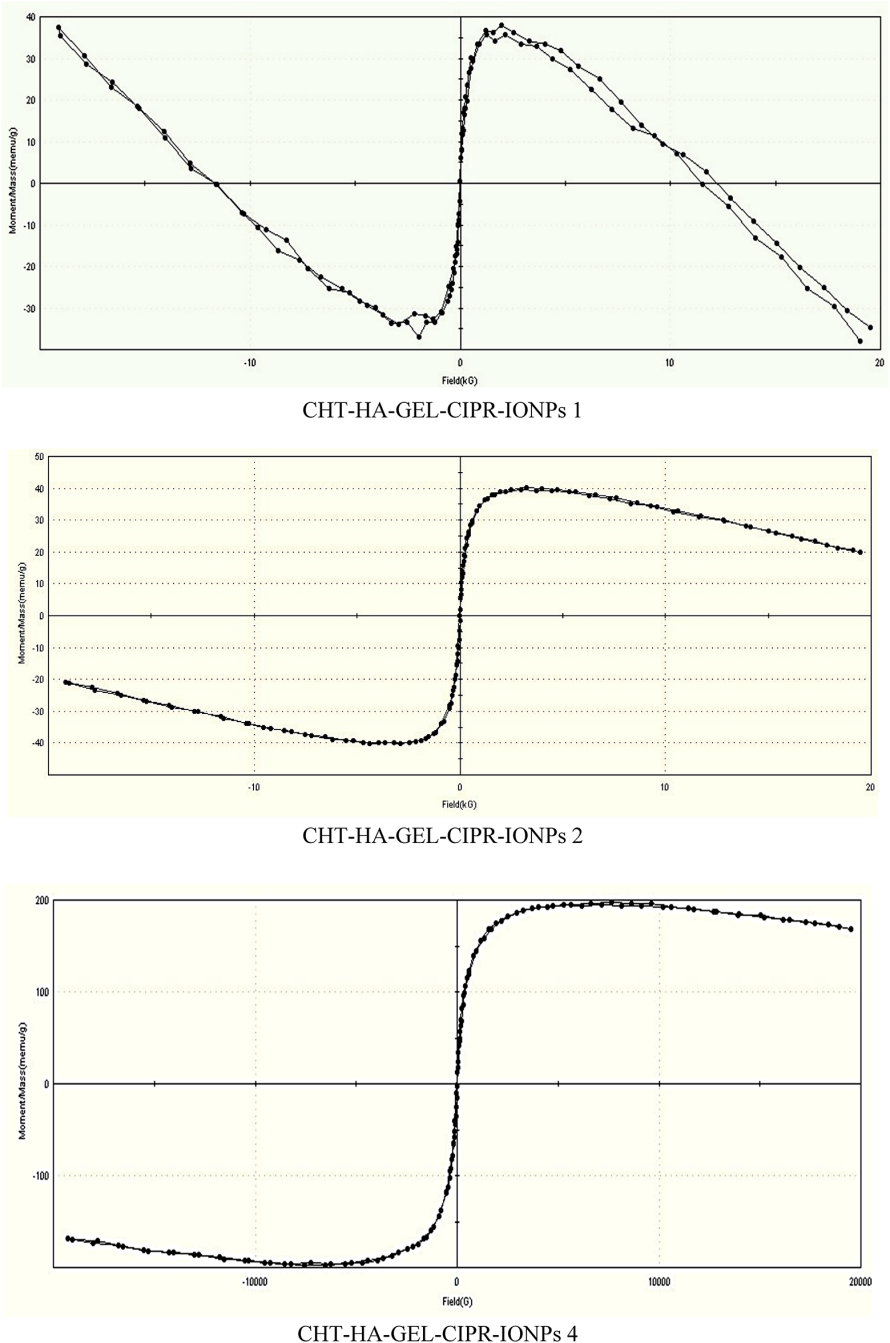
VSM curves of hydrogels formulation loaded with IOPNs and ciprofloxacin.

VSM of prepared freeze-dried CHT-HA-GEL-CIPR-IONPs 0, CHT-HA-GEL-CIPR-IONPs 1, CHT-HA-GEL-CIPR-IONPs 2, and CHT-HA-GEL-CIPR-IONPs 4 are studied by measuring magnetization as a function of field (Fig. 12). The magnetization was recorded in an applied magnetic field of − 20,000 ≤ H (Oe) ≤ 20,000 at room temperature. The hysteresis loop of synthesized nanoparticles is shown in Fig. 12 and the saturation magnetization of the prepared magnetic hydrogels formulations. It is clear from Fig. 12 that all samples have magnetic properties which increase by increasing the amount of IONPs.

#### Drug loading and release

**Fig. 13:**
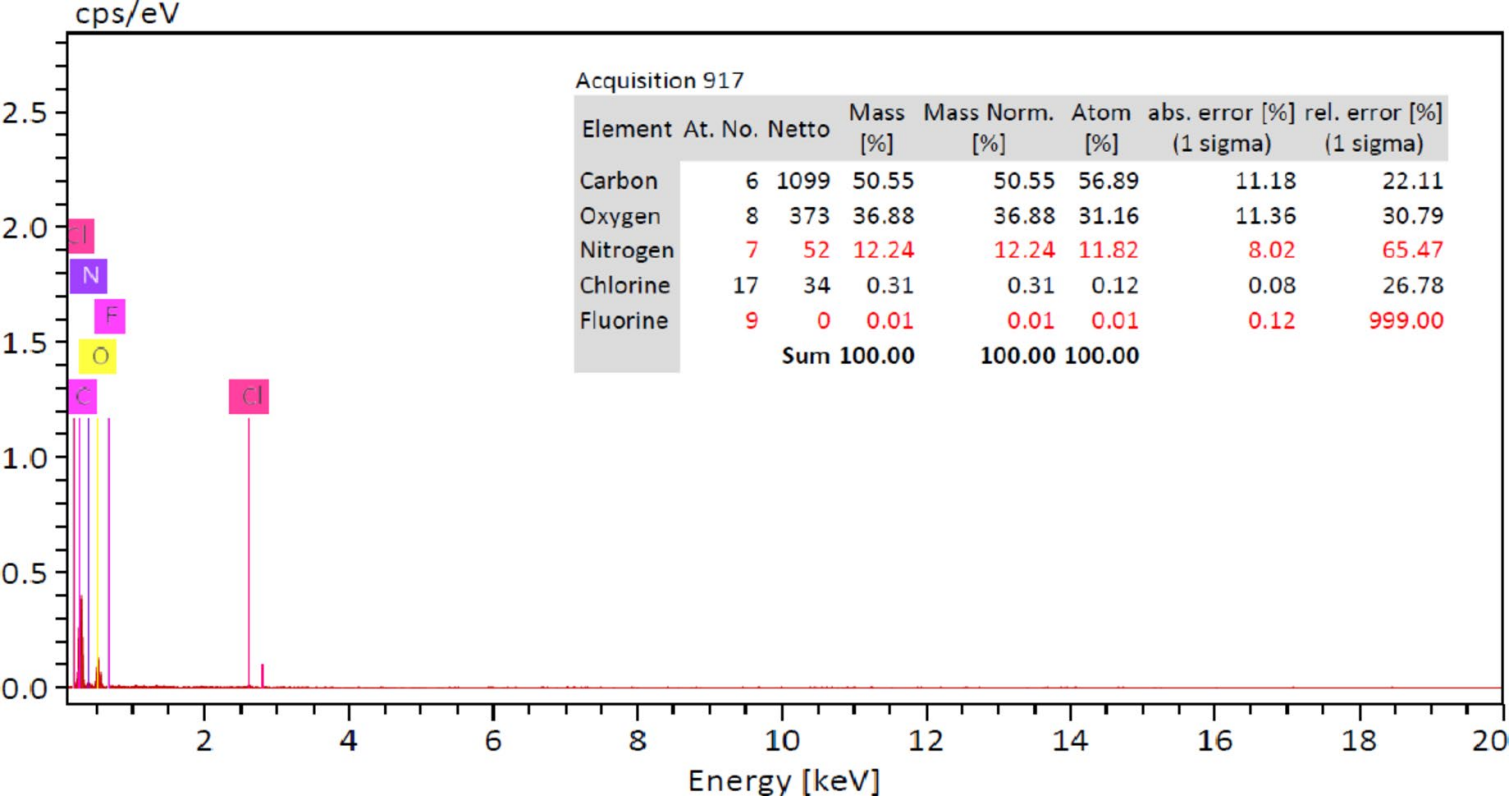
EDX CHT-HA-GEL-CIPR-IONPs 0.

Loading of ciprofloxacin hydrochloride was confirmed by EDX measurement of the loaded freeze-dried hydrogel (CHT-HA-GEL-CIPR-IONPs 0). Figure 13 proved the presence of fluorine in the EDX element analysis along with carbon, hydrogen, oxygen, nitrogen, and sulfur. Presence of fluorine is a quiet proof of presence of ciprofloxacin hydrochloride, as it is the only source of fluorine in the loaded freeze-dried hydrogel formulation (CHT-HA-GEL-CIPR-IONPs 0).

**Fig. 14:**
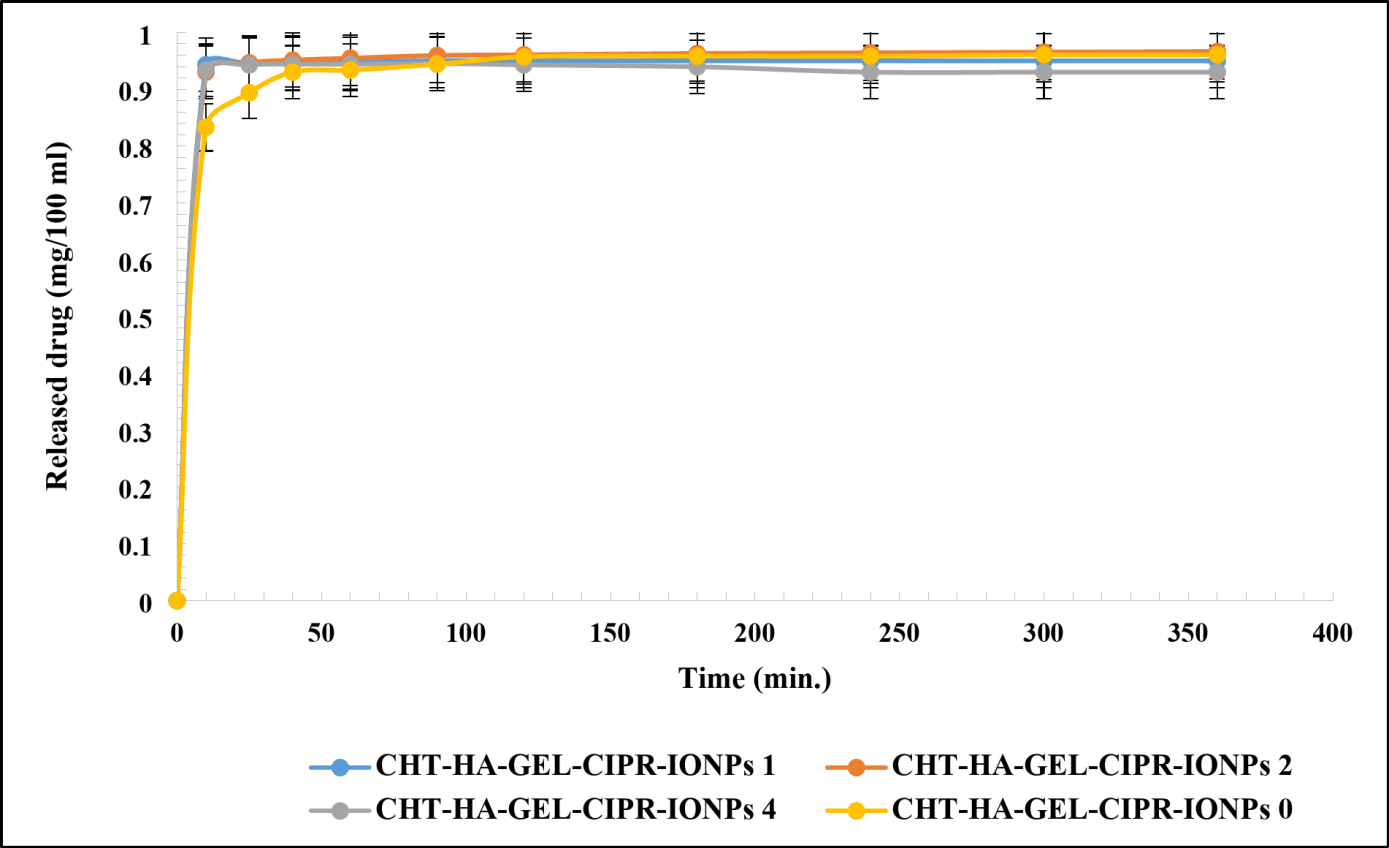
Release profile of ciprofloxacin in magnetic hydrogel.

Figure 14 reveals the release profile of CHT-HA-GEL-CIPR-IONPs 0, CHT-HA-GEL-CIPR-IONPs 1, CHT-HA-GEL-CIPR-IONPs 2, and CHT-HA-GEL-CIPR-IONPs 4 to find out the effect of IONPs on the release profile of ciprofloxacin hydrochloride. Figure 14 showed that all samples have a sustained release of ciprofloxacin hydrochloride over a time about 10 h. It can be seen from Fig. 14 that presence of IONPs has an incremental effect on the release of ciprofloxacin hydrochloride from CHT-HA-GEL-CIPR-IONPs 1, CHT-HA-GEL-CIPR-IONPs 2, and CHT-HA-GEL-CIPR-IONPs 4. That is the presence of IONPs gave a faster release of ciprofloxacin hydrochloride. This phenomenon can be a particularly important in many medical applications.

#### SR %

**Fig. 15:**
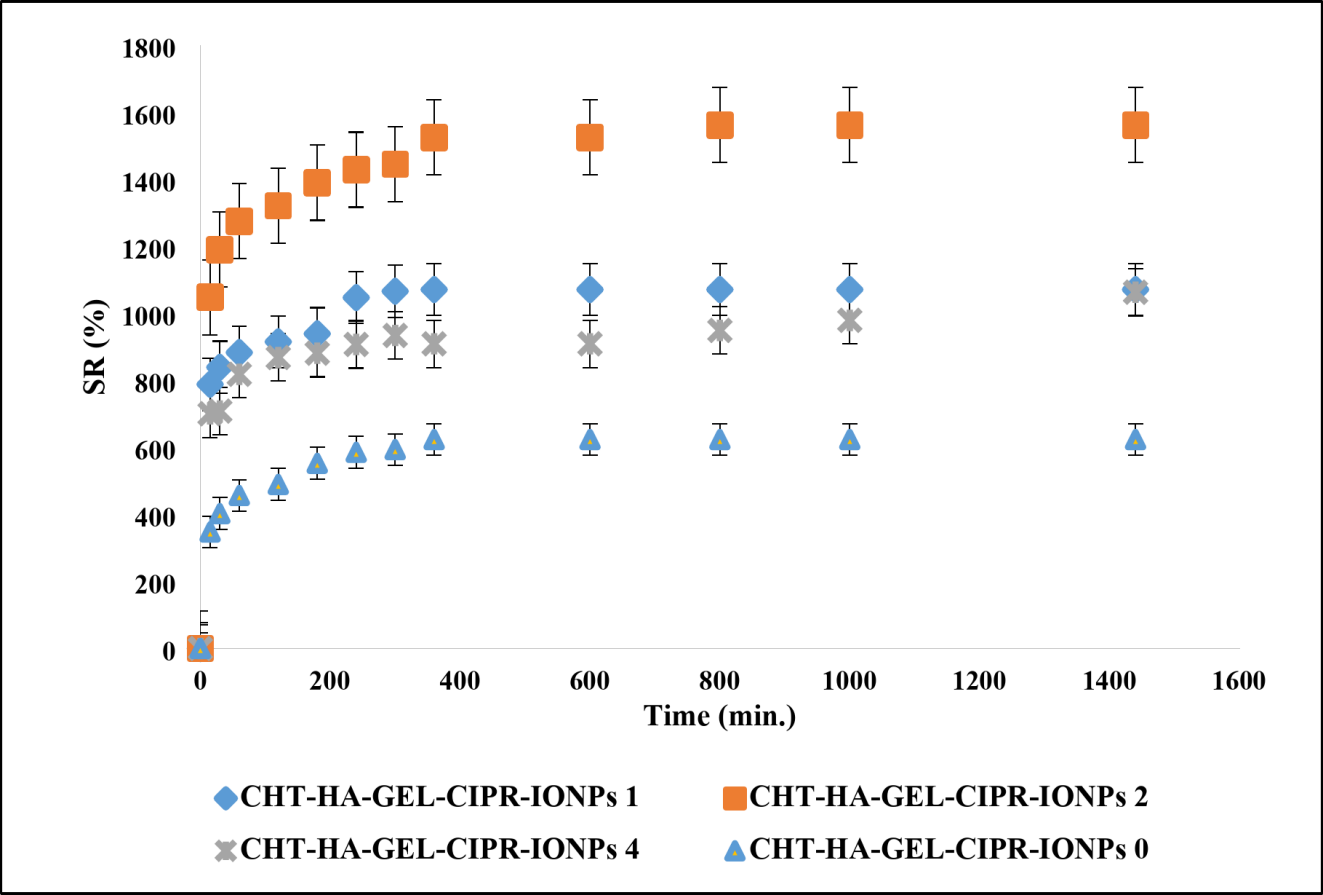
SR % of the prepared hydrogels in presence and absence of MNPs.

SR % of CHT-HA-GEL-CIPR-IONPs 0, CHT-HA-GEL-CIPR-IONPs 1, CHT-HA-GEL-CIPR-IONPs 2, and CHT-HA-GEL-CIPR-IONPs 4 was presented in Fig. 15. SR % of SR % of CHT-HA-GEL-CIPR-IONPs 0, CHT-HA-GEL-CIPR-IONPs 1, CHT-HA-GEL-CIPR-IONPs 2, and CHT-HA-GEL-CIPR-IONPs 4 was increased significantly within the first 40 min of immersion in water, then marginally increased until 24 h of immersion after that the stabilization was achieved as the samples attained its maximum water retention capacity.

#### Antibacterial activity

**Fig. 16:**
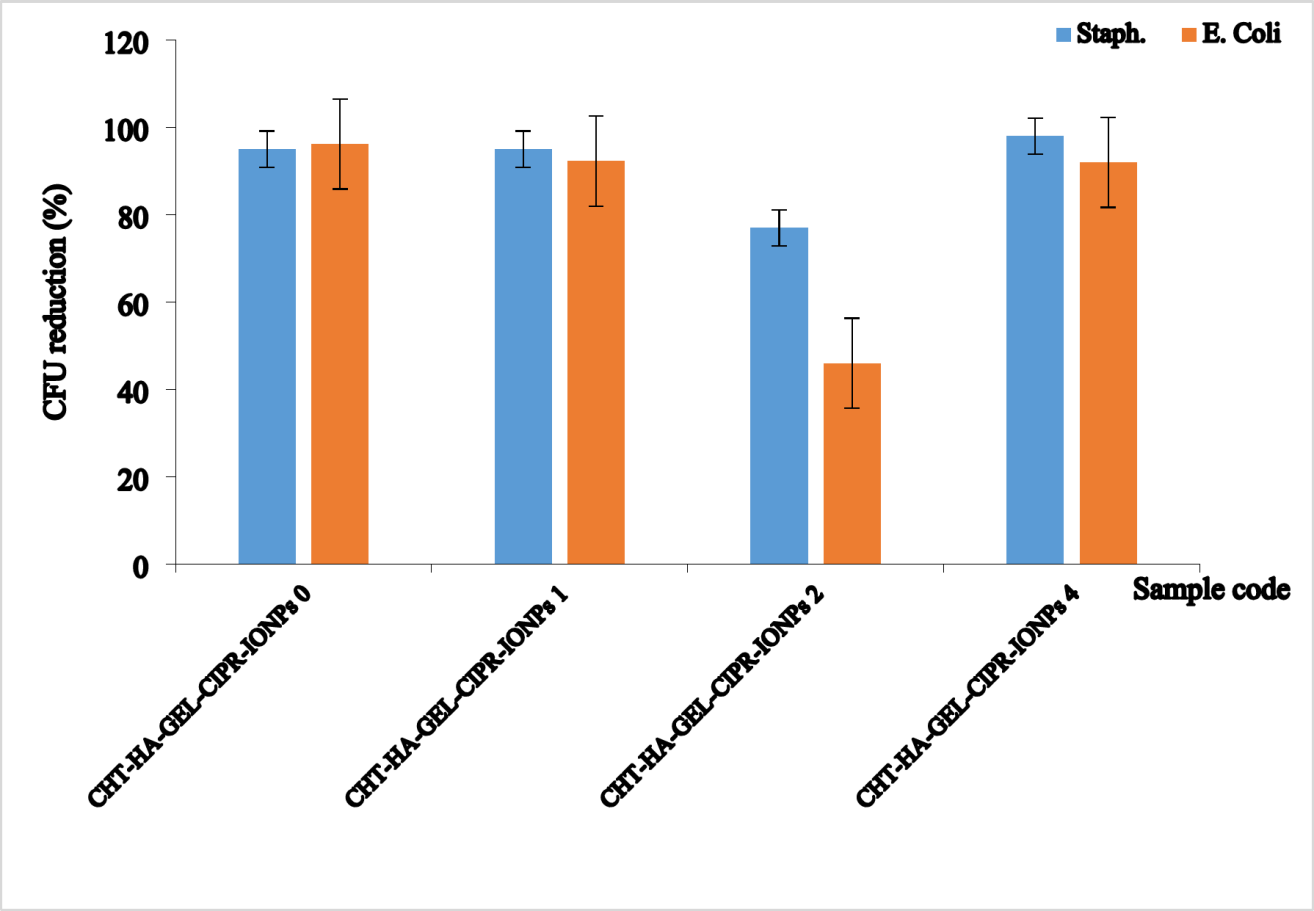
Antibacterial activity of magnetic hydrogels.

The antibacterial CHT-HA-GEL-CIPR-IONPs 0, CHT-HA-GEL-CIPR-IONPs 1, CHT-HA-GEL-CIPR-IONPs 2, and CHT-HA-GEL-CIPR-IONPs 4 was assessed using bacterial reduction percent against *(S. aureus)* and *(E. coli)*, as shown in Fig. 16. All samples have high antibacterial activity against *Gram-positive (S. aureus)* and *Gram-negative* (*E. coli*) bacteria. For *Gram-positive* bacteria (*S. aureus*), the antibacterial activity was ranged from 94% to about 97%.

## Conclusion

Magnetic hydrogels based different volumes of chitosan, 1%, gelatin, 10%, and hyaluronic acid, 1% in presence of glutaraldehyde as crosslinking agent and Fe_3_O_4_ as magnetic nanoparticles were prepared. The hydrogels scaffold and magnetic scaffold hydrogels samples were characterised by scanning electron microscopy (SEM), vibrating sample magnetometry (VSM), and Fourier-transform infrared spectroscopy (FTIR). The porosity, mechanical properties, swelling degree and the antibacterial activity of the hydrogels scaffold were also determined as well as the drug release profiles of ciprofloxacin hydrochloride loaded hydrogels. SEM imaging revealed that the magnetic hydrogels showed a relatively rough morphology with an irregular surface, also SEM showed that. The hydrogel surface has three-dimensional porous microstructures and the porosity varied depending on the hydrogel formulation. The breaking load of hydrogel scaffold increased from 1.361 Kgf to 4.98 Kgf by increasing the glutaraldehyde concentration from 0.2 mL to 0.8 mL. SR % values in water were from 250 to 2000% after 24 h. The antibacterial activity of the hydrogels scaffoldwas ranged from 54% to about 97% for *Gram-positive bacteria* (*S. aureus*) and from about 26–92% for *Gram-negative bacteria* (*E. coli*). The ciprofloxacin hydrochloride loaded hydrogel has a sustained release of ciprofloxacin hydrochloride over a time about 10 h. Presence of IONPs gave a faster release of ciprofloxacin hydrochloride.

## Data Availability

Data is provided within the manuscript.
